# ATP-dependent ligases in trypanothione biosynthesis – kinetics of catalysis and inhibition by phosphinic acid pseudopeptides

**DOI:** 10.1111/j.1742-4658.2008.06670.x

**Published:** 2008-11

**Authors:** Sandra L Oza, Shoujun Chen, Susan Wyllie, James K Coward, Alan H Fairlamb

**Affiliations:** 1 Division of Biological Chemistry and Drug Discovery, Wellcome Trust Biocentre, College of Life Sciences, University of DundeeUK; 2 Departments of Medicinal Chemistry and Chemistry, University of MichiganAnn Arbor, MI, USA

**Keywords:** drug discovery, enzyme mechanism, glutathionylspermidine synthetase, slow-binding inhibition, trypanothione synthetase

## Abstract

Glutathionylspermidine is an intermediate formed in the biosynthesis of trypanothione, an essential metabolite in defence against chemical and oxidative stress in the Kinetoplastida. The kinetic mechanism for glutathionylspermidine synthetase (EC 6.3.1.8) from *Crithidia fasciculata* (*Cf*GspS) obeys a rapid equilibrium random ter-ter model with kinetic constants *K*_GSH_ = 609 μm, *K*_Spd_ = 157 μm and *K*_ATP_ = 215 μm. Phosphonate and phosphinate analogues of glutathionylspermidine, previously shown to be potent inhibitors of GspS from *Escherichia coli*, are equally potent against *Cf*GspS. The tetrahedral phosphonate acts as a simple ground state analogue of glutathione (GSH) (*K*_i_ ∼ 156 μm), whereas the phosphinate behaves as a stable mimic of the postulated unstable tetrahedral intermediate. Kinetic studies showed that the phosphinate behaves as a slow-binding bisubstrate inhibitor [competitive with respect to GSH and spermidine (Spd)] with rate constants *k*_3_ (on rate) = 6.98 × 10^4^ m^−1^·s^−1^ and *k*_4_ (off rate) = 1.3 × 10^−3^ s^−1^, providing a dissociation constant *K*_i_ = 18.6 nm. The phosphinate analogue also inhibited recombinant trypanothione synthetase (EC 6.3.1.9) from *C. fasciculata*, *Leishmania major*, *Trypanosoma cruzi* and *Trypanosoma brucei* with *K*_i_^app^ values 20–40-fold greater than that of *Cf*GspS. This phosphinate analogue remains the most potent enzyme inhibitor identified to date, and represents a good starting point for drug discovery for trypanosomiasis and leishmaniasis.

Chagas’ disease, African sleeping sickness and leishmaniasis (cutaneous, mucocutaneous and visceral) are neglected diseases afflicting millions of people worldwide. All of the drugs used to treat these neglected diseases suffer from deficiencies such as poor efficacy, drug resistance, toxicity or high cost of treatment [1]. The parasitic protozoa causing these diseases belong to the order Kinetoplastida, and comparative genomic and biochemical studies have revealed a number of unique metabolic pathways that are being exploited for drug discovery [2]. One of these involves trypanothione [*N*^1^,*N*^8^-bis(glutathionyl)spermidine] and trypanothione reductase, which replaces not only glutathione/glutathione reductase but also thioredoxin/thioredoxin reductase in mammalian cells [3]. Together with the type I and II tryparedoxin peroxidases [4–6], trypanothione is pivotal in defence against oxidative stress induced by host cell defence mechanisms [7–9] or by redox cycling drugs such as nifurtimox [10,11]. In addition, novel trypanothione-dependent enzymes have been identified, such as trypanothione *S*-transferase [12] and glyoxalase I and II [13–15], that are probably involved in defence against chemical stress. The pertinence of the effects caused by decreasing trypanothione content and thus increased chemical stress highlight the significance of the biosynthetic enzyme(s) of trypanothione as drug target(s) [16].

Trypanothione is synthesized in these medically important parasites from glutathione (GSH) and spermidine (Spd) by a monomeric C-N ligase [trypanothione synthetase (TryS), EC 6.3.1.9], in a two-step reaction with glutathionylspermidine as an intermediate [17–20]. Both trypanothione reductase and TryS have been shown to be essential for parasite survival [21–25]. However, in the insect parasite, *Crithidia fasciculata*, TryS forms a heterodimer with the bifunctional glutathionylspermidine synthetase/amidase (GspS, EC 6.3.1.8/GspA, EC 3.5.1.78) [26]. Previous work suggested that each biosynthetic enzyme independently adds successive molecules of GSH to Spd to make trypanothione [26,27]. However, recombinant TryS from *C. fasciculata* (*Cf*TryS) has been reported subsequently to catalyse both steps of trypanothione synthesis [28]. Although a gene for GspS has not been identified in *Trypanosoma brucei*, there is a pseudogene in *Leishmania major* (accession number AJ748279) [19] and putative genes for GspS within the genomes of *Leishmania infantum* (accession number AM502243) and *Trypanosoma cruzi* (accession number EAN98995) that remain to be functionally characterized. Genome sequencing information has also highlighted the presence of *GSPS* in a range of enteric pathogens such as *Salmonella* and *Shigella* [29,30]. The mechanism and physiological function of this protein are unknown, but in *Escherichia coli* it is proposed to be involved in regulation of polyamine levels during growth [31], and a similar function has been postulated for *C. fasciculata* GspS (*Cf*GspS) [32]. Glutathionylspermidine accumulates only under stationary-phase conditions, and an alternative proposal is that it may be more effective in protecting DNA from oxidant damage than GSH [33]. A previous lack of structural information on this important class of enzymes has been recently resolved with the reported crystal structure of GspS from *E. coli* (*Ec*GspS), which includes the enzyme in complex with substrate, product and inhibitor [34].

Preliminary enzyme characterization has previously been described for *Cf*GspS [35], as well as kinetic studies on the partially purified native enzyme using an HPLC method [36,37]*.* Other studies have identified phosphonic and phosphinic acid derivatives of GSH as moderate inhibitors of *Cf*GspS [38]. The most active of these was a phosphonic analogue of GSH (γ-l-Glu-l-Leu-Gly^P^), which displayed linear noncompetitive inhibition (*K*_i_ ∼ 60 μm). This analogue was further improved as an inhibitor of *Cf*GspS by the substitution of the glycine moiety with amino acid analogues, such as diaminopropionic acid (*K*_i_ ∼ 7 μm) [39]. Although these inhibitors are excellent lead compounds for drug design against the trypanosomatid parasites, none, as yet, has yielded *K*_i_ values in the nanomolar range.

Proteases that catalyse the direct addition of water to proteins or peptides proceed via an unstable tetrahedral intermediate. These enzymes are inhibited by phosphorus-based stable mimics of the intermediate [40]. Such high-affinity analogues are termed transition state analogues or intermediate analogues [41]. Similarly, ATP-dependent ligases involve attack of a nucleophilic substrate on an electrophilic acyl phosphate [42] via a tetrahedral intermediate. These ligases are inhibited by stable analogues of this intermediate [43–45]. Original work on this type of analogue based on glutathionylspermidine was carried out on *Ec*GspS [46,47]. These studies investigated GSH–Spd conjugates (Fig. 1), with the objective of developing enzyme inhibitors that block the biosynthesis of trypanothione [46–51]. The synthetase activity of *Ec*GspS was inhibited by a phosphonate tetrahedral mimic, in a noncompetitive, time-independent manner with *K*_i_ ∼ 10 μm [47], and more potently by the phosphinate analogue in a time-dependent manner with *K*_i_^*^ = 8 nm [46,50]. In each case, the phosphorus-based pseudopeptide had no effect on the amidase activity.

**Fig. 1:**
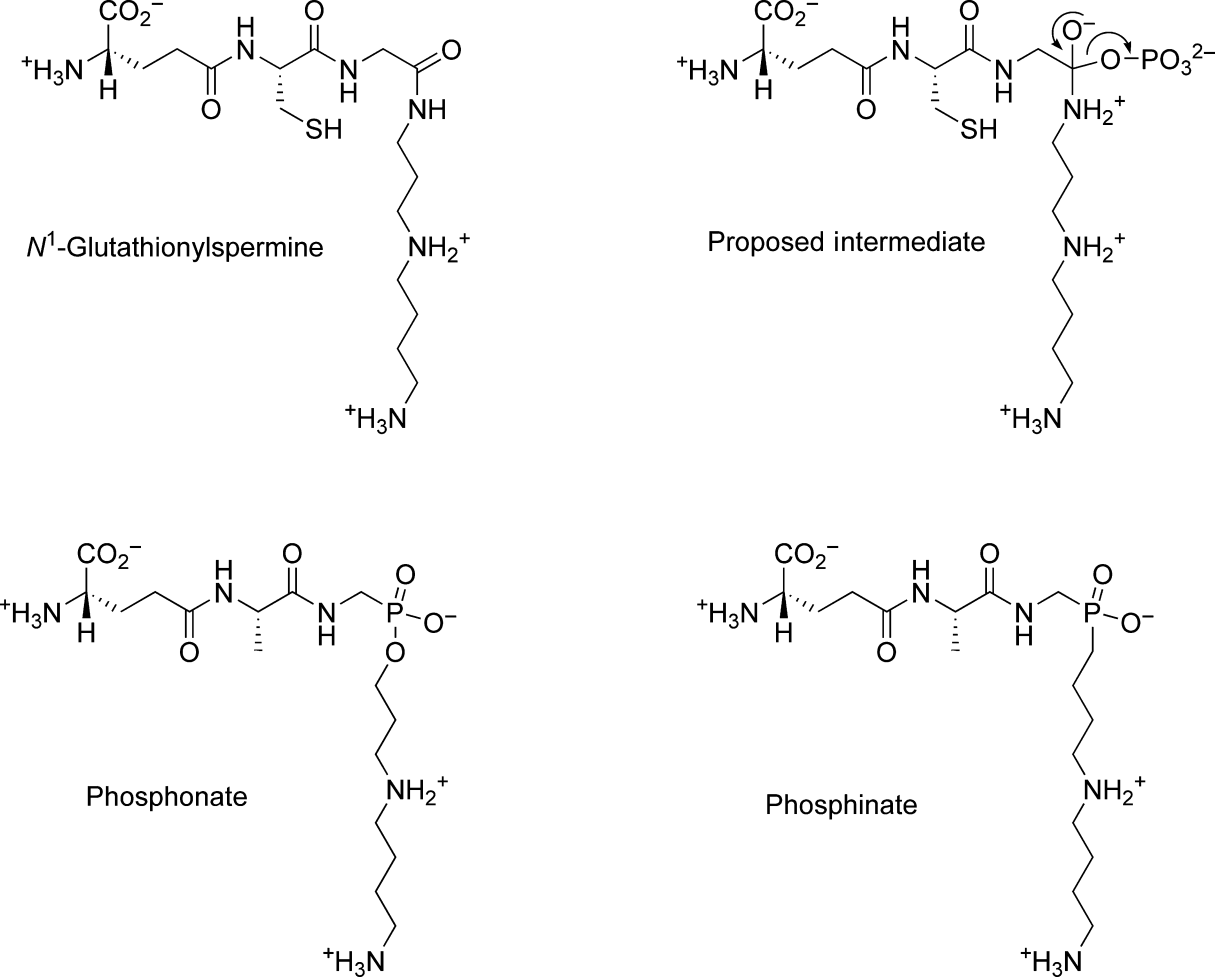
Proposed intermediate of glutathionylspermidine and its phosphon(phin)ate analogues.

Here we examined the kinetic mechanism of *Cf*GspS and determined the modality of inhibition and potency of these compounds against *Cf*GspS and the homologous enzyme TryS from various disease-causing parasites.

## Results

### Initial velocity analysis of the kinetic mechanism of GspS

**Fig. 2:**
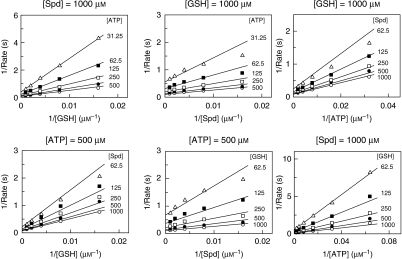
Kinetic analysis of datasets for GspS. Assay details are described in Experimental procedures. The lines represent the global nonlinear fit of the data to the rapid equilibrium random ter-reactant mechanism (Eqn 1) plotted as a Lineweaver–Burk transformation. Reaction rates are reported as catalytic centre activity (s^−1^).

**Fig. 3:**
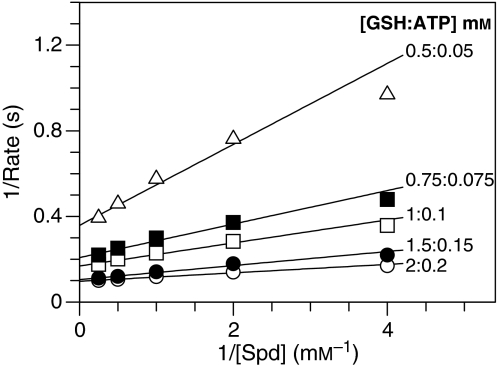
Lineweaver–Burk analysis of the variation of GSH and ATP at a fixed ratio of 1 : 10 versus Spd concentration. The final concentrations of GSH and ATP (mm) for each dataset are displayed on the graph. Reaction rates are reported as catalytic centre activity (s^−1^).

**Fig. 4:**
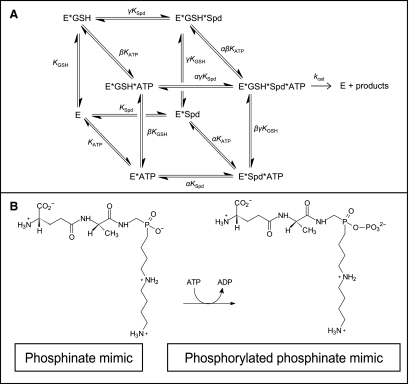
Model of ter-reactant mechanism of GspS catalysis and postulated slow-binding inhibition by the phosphinate mimic. (A) Kinetic mechanism. *K*_GSH_, *K*_Spd_ and *K*_ATP_ are the equilibrium dissociation constants for the binding of substrate to free GspS (E). (B) Postulated structure of the phosphorylated phosphinate mimic.

A matrix of kinetic data was collected in order to determine the kinetic mechanism of GspS. Six families of kinetic data were generated where each ligand (GSH, Spd and ATP) was treated as the varied substrate at different fixed concentrations of another substrate, maintaining a constant saturating concentration of the third substrate [52–55]. The corresponding double reciprocal plots of the data are shown in Fig. 2. A ping-pong mechanism can be ruled out, as the fitted lines of the Lineweaver–Burk plots converge with each combination of substrates. After excluding a ping-pong mechanism, the 16 possible models for rapid equilibrium ter-reactant systems were tested, including random, ordered, and hybrid random–ordered [52]. Statistical tests of each fit revealed that the rapid equilibrium random ter-ter model [see Eqn (1), Experimental procedures] fitted significantly better than any other of the 15 models (*P* < 10^−12^). The interaction factors were close to unity in this model, and when the interaction factors were set *α* = *β* = *γ* = 1, the two fits were not significantly different (*P* > 0.05), but did return ∼ 10-fold lower standard errors for the binding constants. Thus, the simplest model compatible with the data suggests that substrates bind to GspS in any order, without affecting binding of the other substrates, to form a quaternary complex, enzyme–GSH–ATP–Spd. When *α* = *β* = *γ* = 1, the equilibrium dissociation constants for the binding of substrate to the free enzyme are 609 ± 26, 157 ± 5 and 215 ± 8 μm for GSH, Spd and ATP, respectively, and *k*_cat_ = 22.8 ±0.6 s^−1^. When GSH and ATP were varied in a constant ratio (10 : 1) versus various concentrations of Spd, they produced a series of Lineweaver–Burk plots that clearly converged (Fig. 3). This indicates that a product release step does not occur between the binding of ATP or GSH and Spd. Thus, the proposed kinetic model for GspS is consistent with a random ter-reactant mechanism, as shown in Fig. 4A.

### Inhibition by phosphonate analogue

**Fig. 5:**
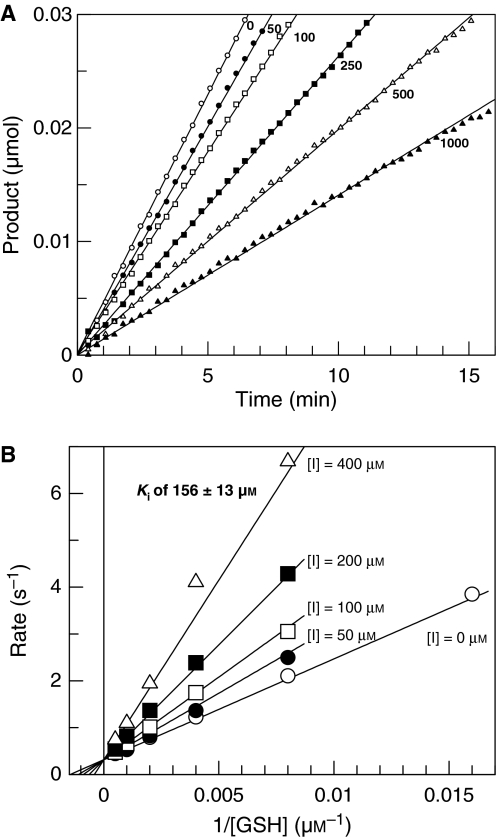
Linear competitive inhibition of GspS by phosphonate analogue. (A) Progress curves demonstrating the classical competitive inhibition of GspS activity by phosphonate. Assays with GspS were performed in 250 μL of assay buffer with 10 nm GspS, 1 mm Spd, 1 mm GSH, 2 mm ATP and various phosphonate concentrations (0–1000 μm) as indicated. The lines fitted to the data points are linear fits for each of the phosphonate concentrations denoted. The linear regression values for all the data points are ≥ 0.997. (B) Kinetic analysis of GspS inhibition by phosphonate. Assays with GspS were performed in 250 μL of assay buffer with 1 mm Spd, various GSH concentrations (62.5–2000 μm), various phosphonate concentrations (50–400 μm) as indicated, and elevated levels of GspS (200 nm). The lines on the Lineweaver–Burk transformation are the best global nonlinear fit of the data to Eqn (2) describing linear competitive inhibition. Reaction rates are reported as catalytic centre activity (s^−1^).

The compounds used in this study were designed to mimic the unstable tetrahedral intermediate formed during GspS-catalysed synthesis of glutathionylspermidine (Fig. 1). However, as reported for *Ec*GspS [47], no time-dependent inhibition of *Cf*GspS was observed with the phosphonate mimic (Fig. 5), which suggests that this compound is not acting as a mimic of the unstable intermediate, but as a bisubstrate analogue [56] incorporating key functional groups of both GSH and Spd in the inhibitor. This compound behaves as a modest classical linear competitive inhibitor of GspS with respect to GSH (Fig. 5B) with a *K*_i_ of 156 ± 13 μm. Note that for classical reversible inhibitors, the rate of product formation is constant provided that there is no significant depletion of substrate or inhibition by product.

### Inhibition by phosphinate analogue

**Fig. 6:**
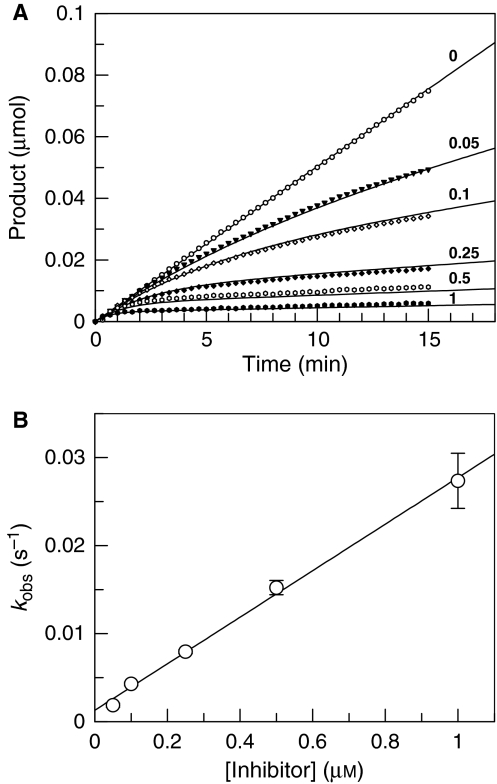
Slow-binding inhibition of GspS by phosphinate analogue. (A) Assays with GspS were performed as described in Experimental procedures with 15 nm GspS, and various phosphinate concentrations (0–1 μm) as indicated, with 1 mm each GSH and Spd. (B) Determination of the association rate *k*_3_′ from the plot of *k*_obs_ as a function of phosphinate concentration. The line represents a linear fit of *k*_obs_ and [*I*] values (phosphinate concentrations). The *k*_obs_ values were calculated from Eqn (4), and the line predicts a slope (*k*_3_′) of 0.026 μm^−1^ s^−1^.

In contrast to the simple, linear inhibition shown by the phosphonate, time-dependent inhibition was observed for the phosphinate mimic (Fig. 6A). In reaction mixtures containing a slow-binding inhibitor initiated by the addition of enzyme, the initial velocity *v*_0_ is independent of inhibitor concentration, but decreases to a slower steady-state velocity *v*_s_ that is dependent on inhibitor concentration [41]. These results are consistent with glutathionylspermidine-dependent phosphorylation of the phosphinate (Fig. 4B), as previously demonstrated for the inhibition of *Ec*GspS [34,46,50]*.* The progress curves for each phosphinate concentration were fitted to Eqn (3) (Experimental procedures) to obtain values for *v*_0_, *v*_s_ and *k*_obs_. Values for *k*_obs_ were then plotted against the inhibitor concentration (Fig. 6B). A linear dependency between [*I*] and *k*_obs_ was observed, and was fitted to Eqn (4) (Experimental procedures) to obtain estimates for *k*_3_′ and *k*_4_. The progress curves used to determine the *k*_obs_ values were obtained at [*S*]/*K*_m_ for GSH of 1.64. The rate constant *k*_3_′ (2.64 × 10^4^ m^−1^·s^−1^) was subsequently corrected for competition by substrate, yielding *k*_3_ = 6.98 × 10^4^ m^−1^·s^−1^ (*k*_3_ = *k*_3_′[1 + [*S*]/*K*_m_]). The *y*-intercept of Fig. 6B yields an estimate of *k*_4_ of 1.3 × 10^−3^·s^−1^. Thus, the overall dissociation half-life for the complex is 0.14 h (enzyme–inhibitor complex half-life values were calculated as the ratio of 0.693/*k*_4_). For an inhibitor of this type, the dissociation constant (*K*_i_) is then obtained from the ratio of *k*_4_/*k*_3_, yielding a *K*_i_ of 18.6 nm. To confirm the *K*_*i*_ value, *v*_0_ and *v*_s_ obtained at different concentrations of inhibitor were fitted to the equation *v*_s_ = *v*_0_/(1 + [*I*]/*K*_i_^app^) by nonlinear regression, yielding a *K*_i_^app^ value of 31.1 ± 2.1 nm, and true *K*_i_ value was calculated to be 19.0 ± 1.3 nm, using the relationship *K*_i_ = *K*_i_^app^/(1 + [*S*]/*K*_m_). Thus both methods of determining *K*_i_ are in excellent agreement.

**Fig. 7:**
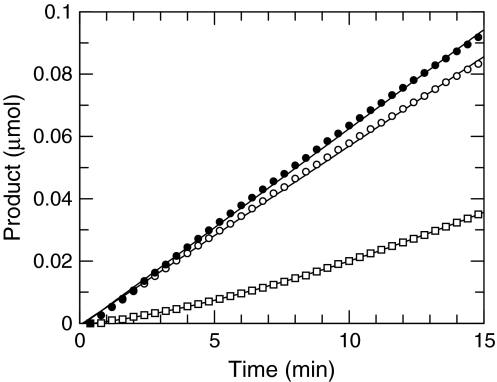
Rate constant (*k*_4_) for dissociation of the GspS–phosphinate complex. Three samples were preincubated for 60 min in 100 mm Hepes (pH 7.3) on ice. The first contained GspS (20 μm) only, the second GspS (20 μm) with excess phosphinate (200 μm) and Mg^2+^-ATP (400 μm), and the third inhibitor/Mg^2+^-ATP only (i.e. no enzyme). All samples were desalted, and the flow-through was added to the coupled assay reaction mix in the following combination: flow-through of sample 1 (enzyme-only control, ○); flow-through of sample 2 (GspS preincubated with excess phosphinate, □); and flow-through of sample 1 plus sample 3 (i.e. a control showing that unbound inhibitor is completely removed using the column method, •), The rate constant associated with the regeneration of enzymatic activity (*k*_4_) was determined as described in the text.

An alternative approach was used to obtain an independent estimate of *k*_4_. In this method, the enzyme was preincubated with excess inhibitor ([*I*] ≥ 10 [*E*]), and the reaction was then initiated with substrate. Under these conditions, a slow release of inhibitor is observed until a steady state is reached. Provided that there is no significant enzyme inactivation, substrate depletion or product inhibition, this steady state should be identical to the steady state established when initiating with enzyme [57]. High concentrations of enzyme and inhibitor were preincubated for 1 h to allow the system to reach equilibrium. Subsequent dilution into a large volume of assay mix containing saturating substrate concentrations causes dissociation of the enzyme–inhibitor complex with regain of activity. Under these conditions, provided that the initial rate *v*_0_ and the effective inhibitor concentration are approximately equal to zero, the rate of recovery of full enzyme activity will provide *k*_4_. When maintaining [*I*] > [*E*] ([*I*] = 250 nm, [*E*] = 20 nm), it proved impossible to measure enzyme activity upon 100-fold dilution into the assay mixture. Instead, high concentrations of inhibitor (200 μm), enzyme (20 μm) and ATP (400 μm) were preincubated on ice for 60 min and then applied to a desalting column to remove all free inhibitor. The following reactions were then analysed: (a) enzyme-only control (Fig. 7, open circles); (b) the complete inhibition reaction, enzyme + inhibitor + ATP (Fig. 7, open squares); and (c) inhibitor-only control added to an equal volume of the enzyme-only control sample (Fig. 7, closed circles). The inhibitor-only control progress curve is linear and matches that of the enzyme-only control, demonstrating that essentially no inhibitor has passed through the resin. The regain of activity experiment (Fig. 7, open squares) clearly shows that an enzyme-bound inhibitor complex passes through the column and undergoes very slow dissociation upon dilution into the assay mixture. Under these conditions, both *v*_0_ and the free inhibitor concentration should be negligible in the final assay, so that the rate of recovery of activity provides the value for *k*_4_. After fitting of the data to Eqn (3) (Experimental procedures), a *k*_4_ value of 1.36 ± 0.06 × 10^−3^·s^−1^ was obtained, in excellent agreement with the value obtained previously by varying the concentration of phosphinate and initiating with enzyme.

### Modality of inhibition

**Fig. 8:**
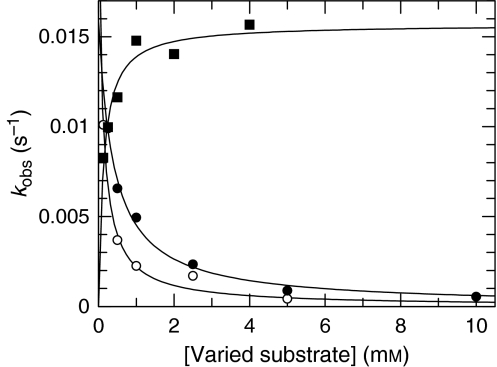
Modality of inhibition by phosphinate analogue. The effect of varying GSH (•), Spd (○) and ATP (▪) on *k*_obs_ was determined at a fixed concentration of phosphinate. The hyperbolic fits were obtained using either Eqn (5) for competitive inhibition (for GSH and Spd) or Eqn (6) for uncompetitive inhibition (for ATP).

The mode of inhibition of the slow-binding phosphinate was determined by examining the effect of varying each substrate on the value of *k*_obs_ at a fixed inhibitor concentration [58]. For a competitive inhibitor, *k*_obs_ decreases in a hyperbolic fashion with increasing concentrations of substrate. This is observed with GSH or Spd as varied substrate (Fig. 8, closed and opened circles). For a noncompetitive inhibitor, *k*_obs_ is independent of substrate concentration (i.e. *k*_obs_ = *k*), whereas for an uncompetitive inhibitor, *k*_obs_ increases in a hyperbolic fashion with increasing concentrations of substrate. As *k*_obs_ increases with increasing concentrations of ATP (Fig. 8, closed squares), this suggests uncompetitive inhibition. These data were then fitted to the appropriate equation for either competitive inhibition [Eqn (5), Experimental procedures] or uncompetitive inhibition [Eqn (6), Experimental procedures]. The respective *K*_m_ values for GSH, Spd and ATP are 400 ± 80 μm, 120 ± 40 μm and 130 ± 26 μm, in reasonable agreement with the respective *K*_m_ values determined directly in the substrate matrix experiment above. Thus, the phosphinate inhibitor behaves as a slow-binding competitive bisubstrate inhibitor with respect to GSH and Spd, but not ATP. The latter observation is consistent with the hypothesis that an electrophilic acyl phosphate is formed by reaction of ATP and GSH. The acyl phosphate then reacts with Spd to form an unstable tetrahedral intermediate, which is mimicked by the stable tetrahedral phosphinate inhibitor. The nucleotide is not a component of the unstable tetrahedral intermediate, and therefore the phosphinate would not be expected to compete with ATP in binding to the enzyme.

To determine whether the phosphinate is turned over by *Cf*GspS in the presence of ATP, the activity of the enzyme (100 nm) was determined in the absence of GSH or Spd plus or minus 1 μm phosphinate over 30 min. After correction for the background rate due to auto-oxidation of NADPH and hydrolysis of ATP in the coupling system, the net rates of endogenous ATPase activity (∼ 0.01% of *k*_cat_) in the presence and absence of inhibitor are 1.4 (± 0.9) × 10^−3^ and 3.0 (± 1.5) × 10^−3^·s^−1^, respectively (mean of three determinations). This shows that the inhibitor is not turned over by the enzyme. However, this method is insufficiently sensitive to detect a single phosphorylation event.

### Inhibition of TryS with phosphinate

Having established that *Cf*GspS is potently inhibited by the phosphinate inhibitor, it remained to be determined whether the homologous enzyme, TryS, could also be inhibited in a similar manner. Owing to the various pH optima, *K*_m_ values for substrates and GSH substrate inhibition profiles of the various TryS enzymes to be compared (*C. fasciculata*, *L. major*, *T. cruzi* and *T. brucei*), a uniform assay was used for IC_50_ determination, i.e. 2 mm Spd, 0.2 mm GSH, 2 mm ATP, 100 mm (K^+^) Hepes (pH 7.2). This allows for direct comparison of the data collected for all the enzymes under conditions that approximate to the physiological metabolite levels found in these organisms [59]. In this study, IC_50_ values, slope factors and *K*_i_^app^ values were determined and found to be 20–40-fold less that that of *Cf*GspS (Table 1). In all cases, the slope factor was approximately 1, indicating simple binding at a single site for all the enzymes tested.

**Table 1:** Inhibition constants of phosphinate against GspS and various TryS enzymes. All assays were performed under conditions approximating to the intracellular physiological state (i.e. pH 7.2, 2 mm Spd, 0.2 mm GSH and 2 mm Mg^2+^-ATP), and were initiated with 100 nm each enzyme in the presence of various phosphinate concentrations. IC_50_ values and slope factors are from the inhibition profiles determined from Eqn (7), and *K*_i_^app^ values were determined from the tight-binding inhibition equation (Eqn 8). The errors represent the standard error of the fit to the appropriate equation.

	Enzyme				
Inhibition constants	*Cf*GspS	*Cf*TryS	*L. major* TryS	*T. cruzi* TryS	*T. brucei* TryS
IC_50_ (nm)	72 ± 6	1380 ± 380	650 ± 25	530 ± 20	1300 ± 50
Slope factor	1.2 ± 0.1	1.1 ± 0.3	1.1 ± 0.04	1.1 ± 0.04	1.1 ± 0.05
*K*_i_^app^ (nm)	29 ± 5	1330 ± 350	580 ± 30	490 ± 20	1200 ± 500

## Discussion

An understanding of the kinetic and chemical mechanism of GspS and TryS involved in the biosynthesis of glutathionylspermidine and trypanothione is crucial for the design of inhibitors against these potential drug targets. TryS is particularly challenging in this respect, as these enzymes display pronounced high substrate inhibition by GSH and form glutathionylspermidine as an intermediate [17–19]. *Cf*GspS does not display substrate inhibition by GSH [35,60], and therefore provides a convenient simple model for this class of ATP-dependent C-N ligases.

The kinetic dataset for *Cf*GspS fits best to a rapid equilibrium random ter-ter reaction mechanism, and definitively excludes a mechanism where either: (a) ADP is released after phosphorylation of GSH prior to binding of Spd; or (b) ADP is released following formation of a phosphorylated enzyme intermediate (ping-pong) prior to binding of GSH or Spd. In this respect, the mechanism for *Cf*GspS is similar to that for γ-glutamylcysteine synthetase from *T. brucei* [53]. However, unlike the case with γ-glutamylcysteine synthetase, we did not detect any marked influence of prior binding of one substrate on the equilibrium dissociation constants of the other substrates [that is, the interaction factors *α*, *β* and *γ* were all close to unity, and statistical analysis did not favour their inclusion in Eqn (1)] (Experimental procedures) [52].

Our results are also broadly in agreement with a previous study which concluded that partially purified *Cf*GspS follows a rapid equilibrium random order mechanism with interaction factors close to unity [37]. However, we were unable to reconcile the peptide sequence data reported by Flohé*et al.* with our own, as it corresponded to our sequence for *Cf*TryS. This discrepancy was later corrected in an erratum by Flohé’s group [36], but raised a second discrepancy concerning *Cf*TryS. In our hands, heterologous expression of *Cf*TryS did not yield active proteins, whereas Flohé’s group reported that authentic *Cf*TryS was able to catalyse the synthesis of trypanothione from GSH, Spd and ATP [28], similar to our findings for TryS from *T. brucei*, *L. major* and *T. cruzi* [17–19]. To resolve this remaining discrepancy, we have repeated our initial study. The newly cloned enzyme was found to differ at position 89, with a serine replacing an asparagine in the original construct (AF006615). The homogeneously pure soluble protein was found to be active with either GSH or glutathionylspermidine, and the product with either substrate was confirmed to be trypanothione by HPLC analysis (data not shown). The reason for our previous failure [27] to detect this activity by heterologous expression in yeast is not apparent, but may have been due to a cloning or PCR error involving this S89N mutation. Nonetheless, we now agree entirely with the report by Comini *et al.* [28] that *Cf*TryS is capable of catalysing both steps in the biosynthesis of trypanothione from GSH and Spd.

A kinetic mechanism has not been determined for the *E. coli* enzyme, but a reaction mechanism has been proposed in which the glycine carboxylate of GSH is initially phosphorylated by the γ-phosphate of ATP to form an acyl phosphate, and this is followed by nucleophilic attack of the *N*^1^-primary amine of Spd on the acyl phosphate, leading to the formation of an unstable tetrahedral intermediate [46,48,49]. Structural studies on *Ec*GspS in complex with substrates and inhibitors provide strong support for this model [34]. Of particular note was the observation that the slow-binding phosphinate inhibitor [46,50] had been phosphorylated by ATP to form the tetrahedral phosphinophosphate in the active site, as previously postulated [51]. In addition, a disordered domain in the apoenzyme was observed to adopt an ordered conformation over the active site when bound with substrates or inhibitor. Our kinetic studies indicate that all three substrates have to bind to the enzyme prior to catalysis. This suggests that formation of the quaternary complex induces closure of the lid domain over the active site to form a catalytically competent complex, thereby preventing access of water to hydrolyse the acyl phosphate intermediate.

Our kinetic analysis shows that the phosphonate analogue displays classical, linear competitive inhibition with respect to GSH, with a modest *K*_i_ of 156 μm against *Cf*GspS, as compared to the mixed-type pattern (*K*_i_ and *K*_i_′ of 6 and 14 μm, respectively) reported for *Ec*GspsS [47]. In contrast, the phosphinate displays slow tight-binding inhibition with a *K*_i_ of 19 nm, similar to the *K*_i_* of 8 nm for the *E. coli* enzyme [46]. Our studies also demonstrate that this inhibitor behaves as a mimic of the unstable tetrahedral intermediate that is proposed to form during the GspS-catalysed reaction as originally postulated [51]. At first sight, the uncompetitive behaviour of the phosphinate inhibitor rather than noncompetitive behaviour is not consistent with a rapid equilibrium random mechanism. However, such an inhibition pattern would be expected if the inhibitor underwent binding followed by a single phosphorylation event, as suggested by the kinetic behaviour observed in this study and others [46,50] and confirmed in the crystal structure of this inhibitor bound in the active site of *Ec*GspS [34]. The glutathionylspermidine phosphinate analogue is also a potent inhibitor of TryS enzymes from *L. major*, *T. cruzi* and *T. brucei*; when assayed under identical conditions approximating to intracellular concentrations, TryS enzymes are approximately 20-fold less sensitive than *Cf*GspS. Although the phosphinate showed no growth-inhibitory activity at 100 μm over 72 h of exposure against *L. major* promastigotes, *T. cruzi* epimastigotes and *T. brucei* procyclics, various chemical modifications could enhance cellular penetration, e.g. acyloxy ester prodrugs [61].

**Fig. 9:**
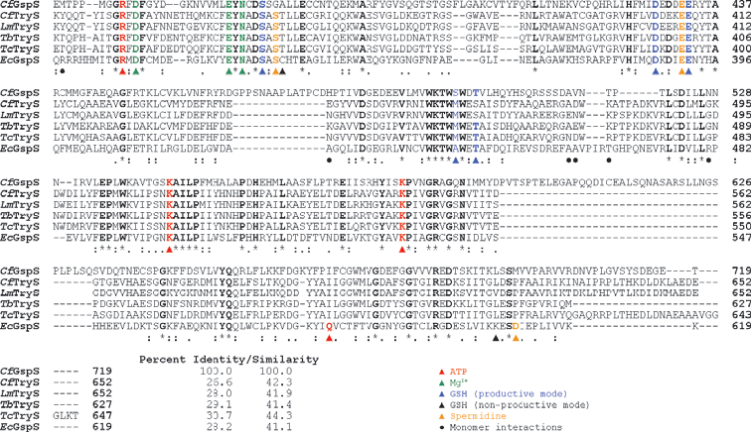
Conservation of key functional residues identified for *Ec*GspS in *Cf*GspS and TryS. The GenBank/EMBL/DDBJ accession numbers used to generate the alignment using t-coffee are: *Ec*GspS (U23148), *Cf*GspS (U66520), *Cf*TryS (AF006615), *L. major* TryS (AJ311570), *T. cruzi* TryS (AF311782) and *T. brucei* TryS (AJ347018). Absolutely conserved residues are marked in bold; coloured residues indicate side chain interactions in *Ec*GspS with substrates or inhibitors [33]. Green triangles, residues involved in binding Mg^2+^; red triangles, three of four residues involved in binding ATP; blue triangles, four of five residues interacting with GSH; yellow triangles, two of three residues implicated in binding of the Spd moiety of the phosphinate inhibitor; black triangles, nonproductive binding mode, where GSH forms a mixed disulfide with Cys338 and an isopeptide bond between the glycine moiety of GSH and Lys607 of the protein; black circles, residues that interact between monomers in *Ec*GspS. Only the relevant C-terminal region of the synthetase domain is shown.

An alignment of *Ec*GspS with *Cf*GspS and other TryS proteins reveals some other interesting features (Fig. 9). First, despite the trypanosomatid proteins having < 30% identity and < 45% similarity, all three residues involved in binding Mg^2+^ (green triangles) and three of four involved in binding ATP (red triangles) are absolutely conserved. Second, four of five residues interacting with GSH (blue triangles) in the productive binding mode are also conserved. Third, two of three residues implicated in binding of the Spd moiety of the phosphinate inhibitor (yellow triangles) are also conserved. Fourth, Pai *et al.* also noted a nonproductive binding mode (black triangles), where GSH forms a mixed disulfide with Cys338 and an isopeptide bond between the glycine moiety of GSH and Lys607 of the protein. However, this is clearly not required for catalysis in the trypanosomatid enzymes, as neither residue is conserved in any of these enzymes. Finally, the *E. coli* enzyme is a homodimer, whereas the trypanosomatid TryS enzymes are monomeric, or heterodimeric in the case of *Cf*TryS and *Cf*GspS. In this case, the residues that interact between monomers in *Ec*GspS (black circles) are hardly conserved at all. One other interesting difference between *Ec*GspS and *Cf*GspS is that the latter enzyme has an additional 100 amino acids. The alignment in Fig. 9 highlights a number of insertions that are dispersed throughout the sequence of *Cf*GspS. These include an insertion of 17 amino acids in the amidase domain and two in the synthetase domain (one of 14 amino acids and the other of 39 amino acids). It may be that these additional insertions in *Cf*GspS are required for its heterodimeric interactions with *Cf*TryS.

From the above analysis, it is not immediately obvious why the phosphinate inhibitor is ∼ 20-fold less potent against the TryS enzymes than against *Cf*GspS and *Ec*GspS. Possibly, the substitution of Asp610, which is involved in recognition of the *N*^8^-amine of Spd, for a proline in TryS (methionine in *Cf*GspS) is a critical factor. Alternatively, the fact that TryS has to accommodate either *N*^1^-glutathionylspermidine or *N*^8^-glutathionylspermidine as well as Spd in the polyamine-binding site may be a significant factor. The current ligand-free structure of *L. major* TryS [62] is not helpful in resolving these issues, and substrates or inhibitors in complex with TryS are needed. In the meantime, the phosphinate inhibitors represent a valuable starting point for further development of drug-like inhibitors against this target.

## Experimental procedures

### Materials

All chemicals were of the highest grade available from Sigma-Aldrich (Gillingham, UK), Roche Diagnostics Ltd (Burgess Hill, UK) or Calbiochem (Merck Biosciences, Nottingham, UK). The phosphonate and phosphinate analogues of glutathionylspermidine were synthesized as previously described [49,51]. The structure and purity of both compounds were confirmed by NMR, high-resolution MS and elemental analysis.

### Expression and purification of GspS

Recombinant GspS was prepared using a 60 L fermenter, and purified to greater than 98% homogeneity as described previously [35], except that a HiLoad Q Sepharose 16/10 column (GE Healthcare, Amersham, UK) was used in the final step. Active fractions were pooled, buffer was exchanged into 100 mm (K^+^) Hepes containing 0.01% sodium azide, 1 mm dithiothreitol and 1 mm EDTA, and the sample concentration was determined using the calculated extinction coefficient of 99 370 at 280 nm. Aliquots of GspS were then flash frozen and stored in aliquots at −80 °C.

### Expression and purification of TryS enzymes

TryS enzymes from *T. brucei*, *L. major* and *T. cruzi* were prepared as described previously [17–19]. In addition, we were able to obtain functionally active *Cf*TryS by generating a new construct in a modified pET15b vector in which the thrombin cleavage site had been replaced by a TEV protease cleavage site. The ORF was PCR amplified from *C. fasciculata* genomic DNA using the sense primer 5′-CAT**ATG**GCG TCC GCT GAG CGT GTG CCG G-3′, which includes an *Nde*I site (underlined) and a start codon (in bold), and the antisense primer 5′-GGA TCC **TTA**CTC ATC CTC GGC GAG CTT G-3′, which includes a stop codon (in bold) and a *Bam*HI site (underlined); the PCR product was subsequently cloned, via pCR-Blunt II-TOPO (Invitrogen, Paisley, UK), into the *Nde*I/*Bam*HI site of pET15bTEV. Sequencing of three independent clones revealed that the sequence was almost identical to the sequence previously deposited for *Cf*TryS (AF006615), except that serine replaced asparagine at position 89 of the ORF. This construct, *Cf*TryS_pET15bTEV, was transformed into BL21(DE3)pLysS-competent cells (Novagen, Merck Biosciences); typically, cultures were then grown in Terrific Broth at 37 °C to *D*_600 nm_ ≥ 1.2, cooled to 22 °C, induced with a final concentration of 0.5 mm isopropyl-β-d-thiogalactoside, and grown for an additional 16 h. Purification of recombinant protein was achieved using two chromatographic steps [5 mL His-Trap (GE Healthcare), TEV protease cleavage (2 h, 30 °C), followed by a HiLoad Q Sepharose 16/10 HP column (GE Healthcare)].

### Assay conditions for the kinetic mechanism of GspS

All kinetic assays were performed at 25 °C using an assay system that couples ADP production to NADH oxidation at 340 nm [35]. Each assay contained 100 mm (K^+^) Hepes (pH 7.3), 0.2 mm NADH, 1 mm phosphoenolpyruvate, 5 mm dithiothreitol or Tris(2-carboxyethyl)phosphine hydrochloride, 0.5 mm EDTA, 10 mm MgSO_4_, 2 U·mL^−1^l-lactate dehydrogenase, and 2 U·mL^−1^ pyruvate kinase (both coupling enzymes were from rabbit muscle, and purchased from Roche), with varying amounts of ATP, GSH and Spd in a total volume of 1 mL. Rates are expressed in moles of substrate utilized per second per mole of enzyme. To determine the kinetic mechanism, data were collected for GspS at a range of substrate concentrations. A complete matrix of rates as a function of substrate concentration (ATP, 31.25–500 μm; GSH, 62.5–1000 μm; and Spd 62.5–1000 μm) was gathered, so that for any given concentration of any one substrate the rates were measured over the entire range of the remaining two substrates. When fixed concentrations of each of these substrates were used, the final concentrations for ATP, GSH and Spd were 0.5, 1 and 1 mm respectively, unless otherwise stated. The assay was initiated by adding GspS (300 nm) and, after a lag of 10 s, the linear decrease in absorbance was monitored for up to 1 min. Data were then globally fitted by nonlinear regression to all possible models for rapid equilibrium ter-reactant systems [52]. The goodness of fit for each model was compared statistically using the *F*-test and kinetic constants obtained by fitting to Eqn (1): 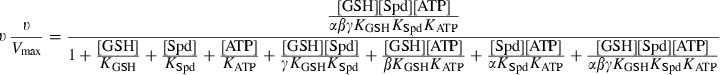
(1)

This equation describes a rapid equilibrium random ter-ter system, where *K*_GSH_, *K*_Spd_ and *K*_ATP_ are the equilibrium dissociation constants for the binding of substrate with free enzyme, and *α*, *β* and *γ* are the interaction factors between Spd and ATP, GSH and ATP, and GSH and Spd, respectively.

### Inhibitors and enzyme inhibition assays

Inhibitor studies employed the coupled assay described above. Possible inhibition of the coupling enzyme system was excluded by substituting glucose and hexokinase for GspS or TryS in the assays, in which case no enzyme inhibition was observed. Reactions were typically carried out as described for the kinetic mechanism, with GSH and Spd (both at 1 mm) and ATP (2 mm) concentrations kept constant. Substrates and inhibitors were preincubated for 10 min before initiation of the reaction with GspS (10–20 nm). Data for the phosphonate analogue were fitted to the Michaelis–Menten equation for competitive inhibition (Eqn 2) when GSH was varied, and analysed using the program grafit: 
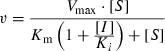
(2)

For time-dependent inhibition by the phosphinate analogue, the progress curves at different inhibitor concentrations can be described by Eqn (3): 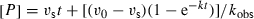
(3) where [*P*] is the product concentration at time *t*, *v*_0_ and *v*_s_ are the initial and final steady-state rates, and *k*_obs_ is the apparent first-order rate constant for the establishment of the final steady-state equilibrium. The resulting values for *k*_obs_ were plotted as a function of inhibitor concentration, *I*, and fitted to Eqn (4) to obtain estimates of *k*_3_′ and *k*_4_: 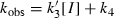
(4)

The rate constant *k*_4_, for the dissociation of the enzyme–inhibitor complex, was also measured directly from the time-dependent recovery of enzyme activity. GspS (20 μm) was preincubated, with or without phosphinate (200 μm) and Mg^2+^-ATP (400 μm), in 30 μL of assay buffer at 4 °C for 1 h, in order to reach equilibrium. A sample containing only inhibitor and Mg^2+^-ATP sample was also included as an internal control to verify efficient retention of the phosphinate by the column. Following preincubation, samples were applied to 0.5 mL of Zeba desalt spin columns (Pierce) and centrifuged to remove unbound inhibitor (1500 ***g***, 2 min, 22 °C). Subsequently, 2 μL of each sample was diluted (1 : 500) into the complete enzyme assay mixture and the absorbance change was monitored. The recovery of enzymatic activity was measured at 340 nm using the coupled assay described above.

To determine the modality of inhibition by the phosphinate, assays were carried out in a reaction mixture of 1 mL containing 1 μm inhibitor in addition to the other components of the coupled assay. When GSH was varied, ATP and Spd were kept constant at 2 and 10 mm respectively; when Spd was varied, ATP and GSH were kept constant at 2 and 10 mm respectively; and when ATP was varied, GSH and Spd were kept constant at 1 mm. The reaction mix was left for 5 min at 25 °C, and the reaction was then initiated with *Cf*GspS (20 nm) and monitored for 15 min. These data were then fitted to the appropriate equation [58] for either competitive inhibition (Eqn 5) 
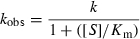
(5) or uncompetitive inhibition (Eqn 6) 
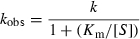
(6) where *k* is the value for *k*_obs_ in the absence of substrate, and *K*_m_ is the binding constant for the varied substrate *S*.

IC_50_ data were also gathered for representative recombinant TryS enzymes (*T. cruzi*, *T. brucei* and *L. major*) [17–19], using more physiological-like conditions, i.e. 2 mm Spd, 0.2 mm GSH, 2 mm ATP, and 100 mm (K+) Hepes (pH 7.2) (the remainder of the components of the coupled assay were as previously above) and various phosphinate concentrations (0–10 μm). Reactions were initiated using 100 nm each enzyme, and the change in absorbance was monitored for 30 min. The resulting steady-state rates were then fitted to the following two-parameter equation (Eqn 7), where the lower data limit is 0, i.e. the data are background corrected, and the upper data limit is 100, i.e. the data are range corrected.


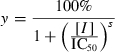
(7)

In this equation, *s* is a slope factor. The equation assumes that *y* falls with increasing [*I*]. The *K*_i_^app^ values of the inhibitor against each enzyme were determined using the following tight-binding inhibition equation [41] (Eqn 8), where the enzyme concentration [*E*] was fixed at 100 nm: 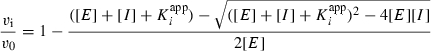
(8)

## Abbreviations

*Cf*GspS: *Crithidia fasciculata* glutathionylspermidine synthetase
*Cf*TryS: *Crithidia fasciculata* trypanothione synthetase
*Ec*GspS: *Escherichia coli* glutathionylspermidine synthetase
GSH: glutathione
GspA: glutathionylspermidine amidase
GspS: glutathionylspermidine synthetase
Spd: spermidine
TryS: trypanothione synthetase
